# Dependence of P-wave dispersion on mean arterial pressure as an independent hemodynamic variable in school children

**Published:** 2013-09-30

**Authors:** Elibet Chávez, Emilio F. González, María del Carmen Llanes, Merlin Garí Llanes, Yosvany García

**Affiliations:** 1 Cardiocentro Ernesto Guevara de Villa Clara. Villa Clara, Cuba.; 2 Universidad Central de Las Villas. Villa Clara. Cuba.; 3 Hospital Pediátrico Universitario José Luis Miranda. Villa Clara.Cuba

**Keywords:** P-wave dispersion, mitral A-wave duration, prehypertension, blood hypertension

## Abstract

**Introduction:**

The relationship between diastolic dysfunction and P-wave dispersion (PWD) in the electrocardiogram has been studied for some time. In this regard, echocardiography is emerging as a diagnostic tool to improve risk stratification for mild hypertension.

**Objective:**

To determine the dependence of PWD on the electrocardiogram and on echocardiographic variables in a pediatric population.

**Methods:**

515 children from three elementary schools were studied from a total of 565 children. Those whose parents did not want them to take part in the study, as well as those with known congenital diseases, were excluded. Tests including 12-lead surface ECGs and 4 blood pressure (BP) measurements were performed. Maximum and minimum P-values were measured, and the PWD on the electrocardiogram was calculated. Echocardiography for structural measurements and the pulsed Doppler of mitral flow were also performed.

**Results:**

A significant correlation in statistical variables was found between PWD and mean BP for pre-hypertensive and hypertensive children, i.e., r = 0.32, *p* <0.01 and r = 0.33, *p* <0.01, respectively. There was a significant correlation found between PWD and the left atrial area (r = 0.45 and *p* <0.01).

**Conclusions:**

We highlight the dependency between PWD, the electrocardiogram and mean blood pressure. We also draw attention to the dependence of PWD on the duration of the mitral inflow A-wave. This result provides an explanation for earlier changes in atrial electrophysiological and hemodynamic characteristics in pediatric patients.

## Abstract (es)

**Introducción:**

La relación entre la disfunción diastólica y la dispersión de la onda P (PWD) en el electrocardiograma se ha estudiado durante algún tiempo. En este sentido, la ecocardiografía se está convirtiendo en una herramienta de diagnóstico para mejorar la estratificación de riesgo en la hipertensión leve.

**Objetivo:**

Determinar la dependencia de las PCD en el electrocardiograma de las variables ecocardiográficas en una población pediátrica.

**Métodos:**

515 niños de tres escuelas primarias fueron estudiados, de un total de 565 niños. Aquellos cuyos padres no quieren que participen en el estudio, así como aquellos con enfermedades congénitas conocidas, fueron los ECG de superficie excluded.12 derivaciones y 4 de presión arterial (PA) se realizó mediciones. Máxima y P-valores mínimos fueron medidos y la discapacidad en el electrocardiograma se calculó. También se realizaron ecocardiografía para medidas estructurales y Doppler pulsado del flujo mitral.

**Resultados:**

Se encontró una correlación significativa en variables estadísticas entre personas con discapacidad y la media de BP para niños prehipertensos e hipertensos r = 0,32, *p* <0,01 yr = 0,33, *p* <0,01, respectivamente. Existe una correlación significativa entre PWD y área de la aurícula izquierda (r = 0,45 y *p* <0,01).

**Conclusiones:**

Se destaca la dependencia de las PCD, el electrocardiograma y la media de la presión arterial. También llamamos la atención a la dependencia de personas con discapacidad en la duración de la entrada de la onda A mitral, así que quizás este resultado proporciona una explicación de los cambios anteriores en las características de electrofisiología auricular y hemodinámicas en pacientes pediátricos.

## Introduction

Atrial Fibrillation (AF) is a paradigm of atrial arrhythmic heterogeneity with an anatomical and electrophysiological complex quality ^1^.

It has been suggested that one of the mechanisms involved in AF pathogenesis is the presence of multiple random re-entrant wavelets that propagate and become extinct or fractionate within the atrial tissue. The correlation between the presence of abnormalities in atrial conduction and paroxysmal AF induction is well documented ^2^. 

Intra- and interatrial conduction prolongation and the homogeneous propagation of sinus impulses are electrophysiological characteristics of patients with paroxysmal AF^3^. AF as a sustained tachyarrhythmia has been associated with both cardiovascular risk factors and a substantial increase in morbidity and mortality. There are many diseases associated with AF, such as heart failure, ischemic heart, valvular heart, thyroid, diabetes mellitus, lung and hypertension. The last infirmity noted is related to all of these diseases^4^.

Concerning hypertension, researchers have demonstrated a significant relationship between changes in the left ventricular geometry and PWD^5^ ^,^ ^6^. These phenomena arise in adults with AF.

The objective of this paper is to determine the association between PWD and mean blood pressure (BP) in children between 8 and 11 years of age.

## Material and Methods

515 school children, between the ages of 8 and 11 years and without known congenital cardiovascular diseases, were studied from 2008 to 2010. Excluded from the study were children whose parents did not want them to be included, and children with congenital cardiovascular diseases, either previously known or diagnosed during the study. 

The main element of the physical examination was the way in which BP measurements were obtained. The conventional method for measurement was used by means of a calibrated oscillometric sphygmomanometer. Measurements were always taken under similar conditions by the same staff utilizing an appropriate-sized cuff with the cuff bladder covering at least 80% of the child's arm. It was measured at the midpoint between the acromion and the olecranon with a pediatric-sized stethoscope^7^.

BP measurements were taken with the child in a seated position with the forearm resting on a table, and after remaining at rest for at least 10 minutes. An unwrinkled cuff was placed at the level of the heart. Four BP measurements were taken on different days. The cuff was inflated to 20 mm Hg above the abolition level of the radial pulse, and it deflated at a rate of approximately 2 mm Hg per second. The systolic BP was determined to be the pressure at which the first sound was heard (Korotkoff phase I) and the diastolic BP were defined by the change of the arterial tone or by the point of noise cessation (Korotkoff phase IV). 

The sample was classified according to the diagnosis of normotensive blood pressure, i.e. with BP below the 90th percentile; pre-hypertensive, by a BP between the 90th and 95th percentiles, and hypertension by a BP higher than the 95th percentile for age, sex and height in all cases. This procedure is consistent with those established in the Fourth Report on the Diagnosis, Evaluation, and Treatment of High Blood Pressure in Children and Adolescents^7^. The mean BP was calculated for each subject and then group averages were determined.

A 12-lead surface electrocardiogram was performed with Nyhom Kodem equipment standardized at a paper speed of 50 mm/second. *P*-waves were measured for each lead. The maximum P value was taken as the highest measured *P*-wave value; the minimum *P* value was noted as the lowest measured value; and the *P*-wave dispersion (PWD) was identified as the difference between the maximum *P* value and the minimum *P* value.

Echocardiography also was performed with Aloka 5000 equipment, using a 3.5 MHz transducer. The existence of structurally normal hearts for the entire sample was confirmed through echocardiography. Mitral inflow was determined by Doppler pulse in an apical four-chamber view. A normal pattern of left ventricular relaxation was identified for all of the 515 children under study. The following echocardiography variables were also obtained: left ventricular diameter (diastolic and systolic diameters), inter-ventricular septum thickness, posterior wall thickness, fractional shortening, ejection fraction, left atrial diameter, velocity and mitral inflow A-wave duration (AWD), respectively given in millimetres per second and milliseconds. These values were used to calculate the left ventricular mass, according to the Deveroux equation^8^.

Left Ventricular Mass (g) = 0.8 x 1.04 x(LVD+ IVS+ WP) x + 0 6 (1)

Where: LVD = left ventricular diameter (mm)

 IVS = inter-ventricular septum (mm)

 WP = posterior wall (mm)

The left ventricular mass index (LVMI) g/cm was calculated by equation 2^9^

LVMI = Left ventricular mass/size ^2^ ^.^ ^7^ (2)

Data were analyzed using the SPSS 17.0 software, and the means and standard deviations were calculated. The differences in continuous variables among 3 diagnosis-related groups and 2 sex-related groups were evaluated by parametric tests. Additionally, a multivariate linear regression was performed to identify the most influential variable from an independent perspective on PWD as the dependent variable. In all cases, a 95% confidence interval was used along with partial correlations for age, weight and height. An ANOVA with the Scheffé test was used to compare means.

## Results

Table 1 shows the mean values for the diagnosis-related groups and their standard deviations. The average PWD increased from 31.85 ms, for normotensive children, to 37.33 ms, for pre-hypertensive children, and up to 39.74 ms in the case of hypertensive children. It showed significant differences between normotensive and pre-hypertensive subjects (*p* < 0.01), as well as between normotensive and hypertensive children (*p* < 0.01). No significant differences were found between pre-hypertensive and hypertensive children (*p *= 0.411). The echocardiographic variables studied included: velocity and mitral inflow Awave duration, left atrium diameter, left and right atrial area and left ventricular mass index. The systolic blood pressure, diastolic blood pressure and mean blood pressure (MBP) were all determined, as were the weight, height, age and body surface area. 

Echocardiographic variables are also shown in Table 1. LVMI mean values between normotensive and pre-hypertensive children showed no significant difference (*p* = 0.118); however, there was a significant difference *(p* = 0.026) between normotensive and hypertensive children. LVMI mean values for pre-hypertensive and hypertensive children were not significantly different (*p* = 0.256). The AWD value decreased from 138.2 ms in normotensive children to 131.6 ms in hypertensive children, and the standard deviation likewise decreased. No significant differences between AWD means were observed in the comparison using an ANOVA with the Scheffé Test and a 95-percent confidence interval (inter-group). 

A multivariate linear regression analysis was also performed with a 95% confidence interval that adjusted for age, weight and height. The purpose was to identify MBP as an independent variable among variables having a greater influence on PWD. Table 1 show that MBP values increased from normotensive to hypertensive (79.6 mmHg for normotensive; 83.5 mmHg for pre-hypertensive, and 87.2 mmHg for hypertensive). A comparison between MBP and diagnostic classifications was also performed. Significant differences in PWD were found between normotensive and pre-hypertensive (*p* < 0.01), normotensive and hypertensive (*p* <0.01), and pre-hypertensive and hypertensive (*p *<0.01). 

**Table 1:**
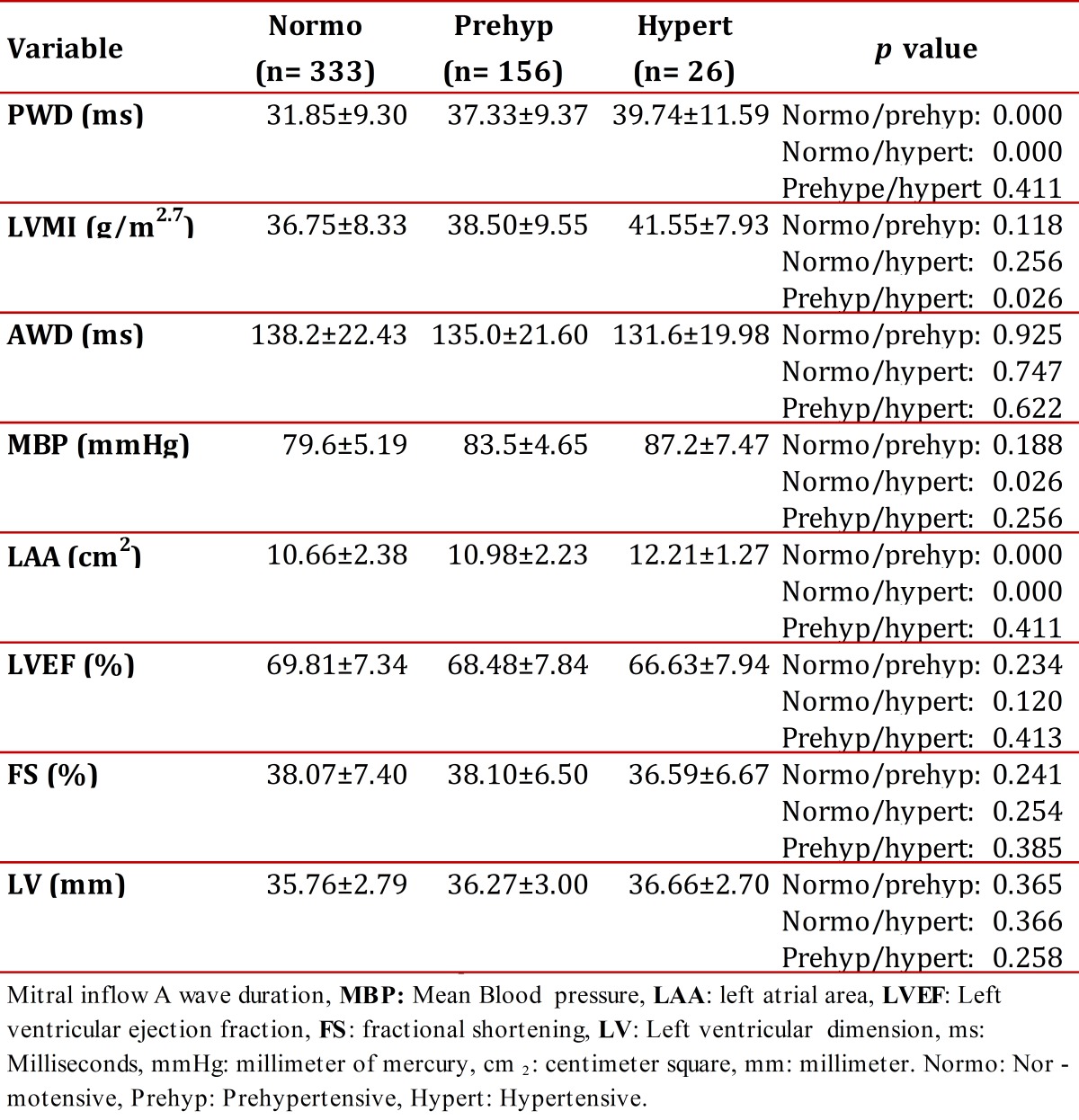
Distribution of mean and standard deviation values for P-wave dispersions and echocardiographic variables

## Discussion

The most important results are the following: a significant correlation in statistical variables was found between PWD and mean BP for pre-hypertensive and hypertensive children (r = 0.32, *p* <0.01 and r = 0.33, *p* <0.01) respectively. There was significant correlation found between PWD and the left atrial area.

There were a high percentage of pre-hypertensive children who had BP values between the 90^th^ - 95^th ^pressure percentiles. Thus, it can be argued that those children are at risk of suffering hypertension in the absence of adequate medical intervention^10^.

The highest PWD in hypertensive and pre-hypertensive groups are shown in Table 1 and indicate an increased vulnerability to the development of atrial arrhythmia. The relationship between this highest PWD and the development of paroxysmal AF by electrophysiological alterations of the left atrial wall has been well documented for adults^2^ ^,^ ^11^. Similar results were obtained for the case of children. A significant difference in the p parameters was found when comparing PWD means for normotensive and pre-hypertensive children, as was the case in comparisons between normotensive and hypertensive children. However, PWD means for pre-hypertensive and hypertensive children were not significantly different. In fact, a similar PWD was evident. This might explain the heterogeneous behavior of the atrial wall in these groups. The highest PWD also showed intra- and inter-atrial delay conduction. This may explain the presence of electrical heterogeneity at this anatomical level^12^. It should be noted that an early hypertension diagnosis, along with the onset of early treatment for these children, would help to lower their cardiovascular risk. Several authors have considered the possibility of returning to normal PWD by using antihypertensive treatment and thus reducing the risk of AF. The latter method would likely help to improve the quality of life for hypertensive patients^13^.

Köse S *et al*.^14^ noticed an increase in LVM secondary to increased BP. They have also pointed out the relationship between LVMI increase/PWD increase/paroxysmal AF in adults with a diagnosis of cardiovascular disease. All of these variables were closely related to this study of children. Therefore, the largest PWD that has been described in adults that is associated with a deteriorating cardiovascular prognosis can be identified from the age of childhood. It may be possible to obtain a regression of PWD in hypertensive children by correctly treating them, as occurs in adult cases.^15^

Changes in left ventricular geometry caused by hypertension, such as occurs in left ventricular hypertrophy (concentric or eccentric) and in LVMI, have been associated with increased PWD. PWD, in particular, has been independently associated with LVMI.^6^ ^,^ ^16^. There is also previous confirmation of the relationship between hypertension and left ventricular hypertrophy and diastolic dysfunction (DD).^16^

DD is frequent in patients with hypertension^17^. A DD study using Doppler pulsed echocardiography showed a decreased AWD in conjunction with increased rates in the pulmonary veins. However, in our sample, the selection criterion was a normal pattern of left ventricular relaxation. We observed the trend toward decreased AWD in hypertensive versus normotensive children, although they did not show significant differences. It has been found that increased systemic pressure leads to increases in left intra-ventricular pressure and retrograde pressures in the left atrium, as part of the transition to DD ^(^ ^17^ ^)^. This knowledge could lead to the pathophysiological argument that despite the absence of altered echocardiographic patterns, this study found a pathophysiological negative influence due to increases in the systemic pressure on left ventricular relaxation. 

Gunduz H, et al^18^ referred to the relationship between DD levels and PWD increases. However, they did not find any relationship to the primary cause of DD. Donoiu *et al*,^19^ however, concluded from their study that the severity of DD in hypertensive children is related to increases in PWD.

Several authors^20^ ^,^ ^21^ who used animal models have described a direct relationship between increases in intra-atrial pressure and the vulnerability of these structures to the development of arrhythmias. In our electrocardiographic study, we observed that high PWD leads to decreased AWD (negative correlation). Therefore, we can argue that from an early age there are pathophysiological influences by increasing systemic BP levels. This leads to impaired ventricular relaxation, despite an E/A normal mitral inflow and subsequent disorder in atrial mechanics. This disorder is represented here by AWD, which corresponds to atrial ejection. The linear regression between PWD and AWD was applied to the diagnosis-related groups, and a significant linear correlation was demonstrated for the entire sample. A significant relationship was not found among normotensive children; however, a significant linear correlation reappeared in pre-hypertensive and hypertensive children, as shown in the relationship between PWD and AWD. 

Beevers G et al^22^ described and defined the pathophysiology of hypertension in which increases in systemic pressure raises the left ventricular pressure and increases the retrograde left atrial pressure. The retrograde left atrial pressure has been related to wall stress and subsequently to the development of electrophysiological conditions that favor the occurrence of atrial arrhythmia^23^. The renin angiotensin aldosterone role in the pathophysiology of hypertension and the remodelled atrial was later described. It has been shown to increase atrial vulnerability and lead to the development of atrial arrhythmia.^24^

Several studies indicate that the relationship between PWD and MBP also guarantees that arrhythmia risk decreases when the BP was normalized. It is shown by the regression of PWD values and decrease of heterogeneity in impulse conduction^2^ ^,^ ^15^. It was noted that this increase in PWD indicates increased atrial vulnerability to the development of atrial arrhythmia. Chávez *et al* ^25^ also described a positive correlation between PWD and the left atrial area in hypertensive children. 

Higher r correlation coefficients and p-wave dispersions can be found in the literature. In our opinion, it is important to consider that these values relate to adults with hypertension, heart failure and other inflammatory secondary changes to the activity of the renin-angiotensin-aldosterone system^ (^ ^24^ ^)^ . We hold the belief that these advanced inflammatory changes are not present in early childhood.

## Conclusions:

We highlighted the dependence between PWD, the electrocardiogram and mean blood pressure and drew attention to the dependence of PWD on the duration of the mitral inflow A-wave. Perhaps these results will provide a satisfactory explanation for the earlier changes in the characteristics of the hemodynamics and atrial electrophysiology in pediatric patients.
